# Ladder-like Poly(methacryloxypropyl) silsesquioxane-Al_2_O_3_-polybutadiene Flexible Nanocomposites with High Thermal Conductivity

**DOI:** 10.3390/gels9100810

**Published:** 2023-10-10

**Authors:** Pietro Mingarelli, Chiara Romeo, Emanuela Callone, Giulia Fredi, Andrea Dorigato, Massimiliano D’Arienzo, Francesco Parrino, Sandra Dirè

**Affiliations:** 1 Department of Industrial Engineering, University of Trento, Via Sommarive 9, 38123 Trento, Italychiara.romeo@unitn.it (C.R.); emanuela.callone@unitn.it (E.C.); giulia.fredi@unitn.it (G.F.); andrea.dorigato@unitn.it (A.D.); 2 “Klaus Müller” Magnetic Resonance Laboratory, University of Trento, Via Sommarive 9, 38123 Trento, Italy; 3 Department of Materials Science, INSTM, University of Milano-Bicocca, Via R. Cozzi 55, 20125 Milano, Italy; massimiliano.darienzo@unimib.it

**Keywords:** ladder-like silsesquioxanes, sol-gel, photocurable gels, alumina, surface functionalization, polybutadiene, nanocomposites, thermal conductivity

## Abstract

Ladder-like poly(methacryloxypropyl)-silsesquioxanes (LPMASQ) are photocurable Si-based gels characterized by a double-stranded structure that ensures superior thermal stability and mechanical properties than common organic polymers. In this work, these attractive features were exploited to produce, in combination with alumina nanoparticles (NPs), both unmodified and functionalized with methacryloxypropyl-trimethoxysilane (MPTMS), LPMASQ/Al_2_O_3_ composites displaying remarkable thermal conductivity. Additionally, we combined LPMASQ with polybutadiene (PB) to produce hybrid nanocomposites with the addition of functionalized Al_2_O_3_ NPs. The materials underwent thermal stability, structural, and morphological evaluations via thermogravimetric analysis (TGA), scanning electron microscopy (SEM), energy dispersive X-ray spectroscopy (EDXS), Fourier transform infrared spectroscopy (FTIR), and solid-state nuclear magnetic resonance (NMR). Both blending PB with LPMASQ and surface functionalization of nanoparticles proved to be effective strategies for incorporating a higher ceramic filler amount in the matrices, resulting in significant increases in thermal conductivity. Specifically, a 113.6% increase in comparison to the bare matrix was achieved at relatively low filler content (11.2 vol%) in the presence of 40 wt% LPMASQ. Results highlight the potential of ladder-like silsesquioxanes in the field of thermally conductive polymers and their applications in heat dissipation for flexible electronic devices.

## 1. Introduction

Silsesquioxanes (SSQs) are a family of hybrid organic-inorganic gels where each silicon atom is linked to three oxygen atoms and connected to an organic function or the hydrogen atom [1]. Two main classes of SSQs display a tunable architecture with defined functional properties [2]: polyhedral oligomeric silsesquioxanes (POSS) and ladder-like polysilsesquioxanes (LPSQ). POSS present oligomeric cage structures with an inorganic silica-like core (Si_8_O_12_) surrounded by eight covalently bonded organic groups (R_8_), while a typical LPSQ is a polymeric analog with a linear, double-strained siloxane backbone, where each Si atom is linked to an organic side-chain [3,4,5,6,7,8,9,10]. The sol-gel chemistry provides a straightforward route to create silsesquioxanes. This method presents extensive opportunities to fabricate smart and functional materials by leveraging the stability of the Si-C bond as well as the wide array of available organotrialkoxysilanes [1]. Moreover, ladder-type silsesquioxanes are characterized by a useful combination of properties such as mechanical and thermal stability, high compatibility with polymers, and good solubility in many organic solvents [3,11,12,13].

LPSQs with methacryloxypropyl side-chains, namely ladder-like poly(methacryloxypropyl) silsesquioxanes (LPMASQ), are gels that can be easily photocured upon thermal or light-induced activation, thus providing controllable and tunable elasticity or hardness [14,15]. This feature, along with the abovementioned outstanding properties typical of SSQs, recently raised the attention of the scientific community on LPMASQ [15,16]. Our group has recently employed LPMASQ as a molecular filler in polybutadiene-based nanocomposites [14,17] and demonstrated that adjusting the thermal, dielectric, and mechanical properties of this polymer represents a key point for many large-scale applications. D’Arienzo et al. showed that LPMASQ, dispersed in polymer matrices at loadings up to 10 wt%, organizes itself in domains with stacked microstructures, thereby inducing peculiar dielectric properties and enhancing mechanical performance. Structural analyses also revealed reduced mobility of PB chains near the hybrid interface due to weak interactions between PB and LPMASQ domains [14]. These interactions underlie the thermal and mechanical stabilization effects induced by SSQs within the PB matrix [18]. The discovery of the stacked structures induced the same group to use LPMASQ as a layered nanofiller in PB-based active-passive oxygen-scavenging nanocomposites. TiO_2_ nanoparticles were used as the active component with the capacity to photo-activate oxygen and capture it in the polymeric matrix, while LPMASQ served as the passive component, impeding oxygen diffusion through the material due to the tortuous path formed by its stacked organization [17].

The structured nature of LPMASQ and its ability to limit the movement of PB chains imply potential applications for this material in the realm of thermally conductive nanocomposites. Moreover, the flexibility, low density, ease of processing, design freedom, electrical insulation, and mechanical and thermal stability of both LPMASQ and PB-LPMASQ nanocomposites make them promising starting materials for synthesizing thermally conductive nanocomposites for advanced electronic applications such as optoelectronics, photonics, integrated circuits, and computer electronics [19,20,21,22,23]. In fact, the increasing power density and decreasing size of these new technologies require effective heat transfer in order to maintain safe operating temperatures, thus ensuring reliable performances and extending their lifetime [24,25]. Notably, even if traditionally used due to their high thermal conductivity, metals possess relevant disadvantages (high electrical conductivity, corrosion issues, high density, and limited design possibilities), which strongly limit their use for these advanced applications [26]. On the other hand, as in the case of most polymers, elastomers such as silicon rubber and PB intrinsically possess low thermal conductivities, far lower than the target values required [27,28,29]. Even though several theoretical studies highlighted the effects of tuning intrinsic features of PB [30], it is imperative to introduce high thermally conductive fillers into the polymeric matrix to provide suitable materials for thermal management [19,31]. Considerable scientific efforts have been devoted to this field by using ceramic fillers such as silicon carbide (SiC) [32,33], silicon nitride (Si_3_N_4_) [34], boron nitride (BN) [35,36], aluminum nitride (AlN) [37,38], zinc oxide (ZnO) [39,40,41], and alumina (Al_2_O_3_) [42,43,44]. Out of these materials, alumina is widely used as a filler despite its lower thermal conductivity (ranging from 30 to 38 W m^−1^ K^−1^) compared to other ceramic fillers. In fact, alumina is cheap, non-toxic, and can be easily obtained in various sizes and shapes [45]. Generally, achieving the desired enhancement in the thermal conductivity of rubber nanocomposites requires high Al_2_O_3_ loadings and small particle sizes [46,47]. However, this can lead to issues such as viscosity increase and filler agglomeration, which can ultimately undermine the mechanical performance of the nanocomposite. Therefore, nanometric, surface-functionalized alumina particles have been proposed to enhance the affinity with the polymeric matrix [48]. Nanosized fillers generally require lower concentrations to form a preferential path for heat transfer in the composite, providing additional benefits such as the flexibility to adjust the transparency of the final nanocomposite [49]. However, the use of nanofillers leads to high interface density within the composite, with the undesired effect of increasing the interfacial thermal resistance (ITR) caused by discontinuities along the path of phonons active in thermal transport [50,51]. Nanofiller functionalization, generally used to improve filler dispersion and reduce viscosity [49], can also promote covalent or weak interactions between the composite’s phases, and therefore, it is expected to facilitate thermal transport amongst the different phases [52,53,54,55,56]. To this purpose, polymer blends have been explored as matrices for composites [54,57,58,59], wherein one of the phases possesses a particular affinity to the filler.

Recently, Mirizzi et al. [44] recounted the synthesis of PB nanocomposites containing alumina nanoparticles decorated with POSS. In this process, SSQ units that were covalently grafted to the alumina surface played a vital role in promoting filler-matrix compatibility, leading to a uniform distribution and a continuous network of alumina nanoparticles. These features provided a relevant increase (60–70%) in thermal conductivity compared with the analogous composites containing bare alumina, even at low alumina loadings. These results encouraged us to investigate the effect of ladder-like SSQs (LPMASQ) addition instead of POSS. In fact, the behavior of cages and ladder-like units has been compared in the literature to showcase the role of SSQ structural organization [2].

Given the already demonstrated mechanical reinforcement effect of LPMASQ in the PB matrix and the conceptual background described above, this work aims to investigate the thermal conductivity of nanocomposites comprised of LPMASQ and variable amounts of bare and functionalized alumina NPs and their blends with PB matrices. In detail, alumina nanoparticles were functionalized with methacryloxypropyl trimethoxysilane (MPTMS), the same organosilane used for LPMASQ synthesis, to produce LPMASQ-alumina nanocomposites with high filler loadings. Then, the affinity between LPMASQ and functionalized alumina was exploited to produce composites in which the polymeric matrix was obtained by blending LPMASQ with PB. The structural and microstructural properties of nanocomposites prepared by solution casting and photo-curing were investigated using the FTIR, multinuclear solid-state NMR, and SEM-EDXS techniques. The thermal behavior was studied by thermogravimetric analysis, and the thermal conductivity was measured by light flash analysis (LFA).

## 2. Results and Discussion

### 2.1. Ladder-like Polymethacryloxypropyl Silsesquioxane/Alumina Nanocomposites

#### Structural and Morphological Characterization

LPMASQ/alumina nanocomposites were prepared by adding bare and MPTMS-functionalized alumina nanoparticles to ladder-like LPMASQ gels. Samples are labeled as XA_L or XAF_L, where X denotes the weight percentage of A (bare alumina) or AF (functionalized alumina) with respect to LPMASQ (indicated by L) (Section 4, Table 2). Flexible and quite transparent films, free of macroscopic defects, were obtained by solution-casting and photo-curing. The thickness of the films ranged from 200 to 400 μm. Table 2 lists the labeling and filler volume percentage of the prepared nanocomposites.

LPMASQ gels were synthesized by sol-gel chemistry (Scheme S1) according to an established procedure [14,16,17,60,61], and the synthesis reproducibility was checked by infrared and NMR spectroscopies (Supplementary Materials Figure S1a–c). The FTIR spectrum shows the characteristic siloxane stretching bands of ladder-like silsesquioxanes at 1100 and 1020 cm^−1^ [1,14,15,16,17]. The high intensity of the band at 1020 cm−^1^ is an indication of some structural irregularity [15,17]. Carbonyl and vinyl stretching vibrations of the methacrylate groups are detected at 1712 and 1637 cm^−1^, respectively; a very weak band centered at 3500 cm^−1^ reveals the presence of few uncondensed silanols, in agreement with about 7% terminal T^2^ units (according to the usual NMR labeling, T^n^ represents SiCO_3_ units and n is the number of bridging oxygens) observed in the ^29^Si NMR spectrum (Figure S1c) [16,17].

**Figure 1:**
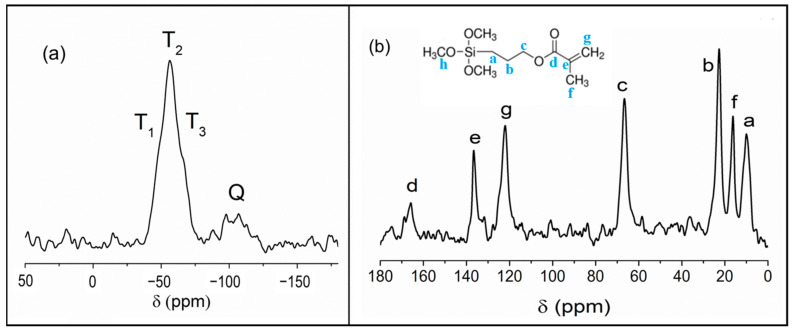
(**a**) ^29^Si and (**b**) ^13^C CPMAS NMR spectra of functionalized nanoparticles. Labeling of Si units is shown in the ^29^Si spectrum; in the ^13^C spectrum, peaks are labeled according to the MPTMS structure shown in the inset.

The effective functionalization of commercial alumina nanoparticles with MPTMS (Scheme S2) was assessed by solid-state nuclear magnetic resonance (NMR). The ^29^Si CPMAS NMR spectrum (Figure 1a) shows the band produced by the overlapping of T^1^, T^2^, and T^3^ resonances, respectively, at −47, −56, and −65 ppm, due to the organosilane grafted onto the particle surface. The weak band at −100 ppm, attributed to Q units, is due to silica present in the commercial alumina sample, as confirmed by the ^29^Si spectrum recorded on bare alumina. In the ^13^C CPMAS NMR spectrum (Figure 1b), the absence of -OCH_3_ resonance at 50 ppm (carbon h in the scheme of Figure 1b) demonstrates the complete hydrolysis of the alkoxide groups, and the downfield shift of methylene carbon (a) of the propyl chain compared to pristine MPTMS is an index of organosilane condensation [10], in agreement with ^29^Si NMR results. The presence of the resonances at 122, 132, and 166 ppm, respectively, attributed to olefinic and carbonyl carbons, and the absence of signals related to the polymerization of the methacrylate group confirmed the maintenance of the reactive end-chain function [10].

TGA analyses on bare and functionalized alumina NPs were recorded to evaluate the yield of the functionalization reaction (Figure S2). The amount of grafted MPTMS was calculated from the mass loss ranging from 150 to 800 °C, as described in the Supplementary Materials. The net weight loss of functionalized alumina NPs compared with bare alumina was 1.7 wt%, corresponding to a grafting density of 1.46 molecules · nm^−2^, calculated according to Equation (S1). This value is quite low compared to the degree of functionalization obtained with the same organosilane in the case of amorphous mesoporous silica particles [16] and can be ascribed to the poor surface reactivity of crystalline alumina nanoparticles.

**Figure 2:**
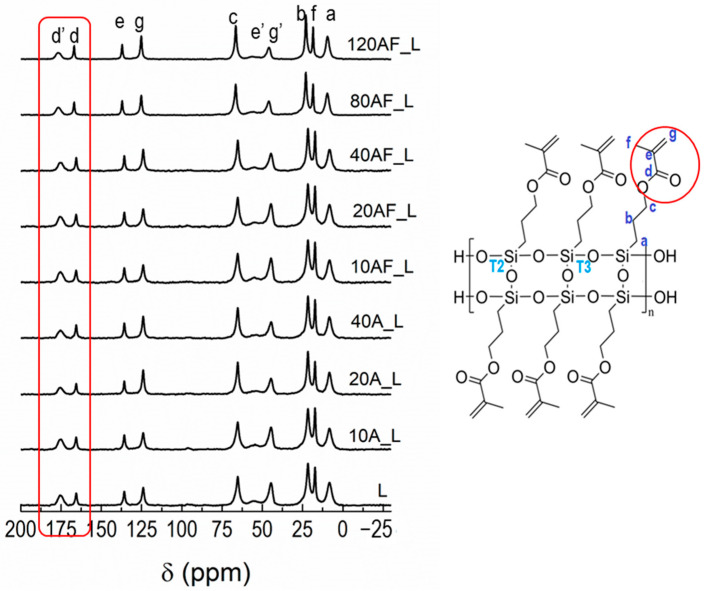
^13^C CPMAS NMR spectra of photo-cured nanocomposites; LPMASQ gel structure with carbon labeling is also shown.

LPMASQ/Al_2_O_3_ nanocomposites were prepared by UV-curing (Scheme S3), and the effect of alumina NPs on the polymerization ability of ladders methacrylate groups was investigated by ^13^C CPMAS NMR (Figure 2). The spectra of the photopolymerized samples display the resonances due to the functional groups of organic side-chains observed in pristine LPMASQ (Figure S1b) [14,15,16,17] with the reduction in intensity of olefinic carbons (e and g) and additional resonances due to the aliphatic carbons (e’ and g’) created by vinyl polymerization. In addition, a second carbonyl resonance (d’) can be observed, which is close to e’ and g’ and downfield shifted with respect to the one present in unreacted LPMASQ (d) in consequence of the increased structure rigidity upon polymerization [14]. All the other resonances do not experience any detectable shift. The extent of polymerization of methacrylate functions can be evaluated by the ratio between the integration area of d’ and the sum of the d and d’ areas [15]. Table S1 presents the polymerization degree of the nanocomposites produced as a function of the filler content. Regardless of the surface functionalization, methacrylate polymerization ability reduces with increasing filler loading, consistent with earlier findings on LPMASQ nanocomposites housing silica and titania NPs [15,16,17]. The decrease in the degree of polymerization is slightly more evident in the case of bare particles since the methacrylate groups on the surface of functionalized particles can participate in polymerization with both the matrix and exposed groups on other NPs.

In agreement with recently reported results [15], photo-induced LPMASQ crosslinking did not affect the integrity of the ladder-like structure according to the silicon-29 spectrum, which highlights an almost unchanged lineshape and chemical shift of the T^3^ resonance (Figures S1c and S3) and a small decrease in T^2^ units (to 4%), in accordance with negligible variations observed in the infrared siloxane asymmetric stretching band of the photo-cured samples.

**Figure 3:**
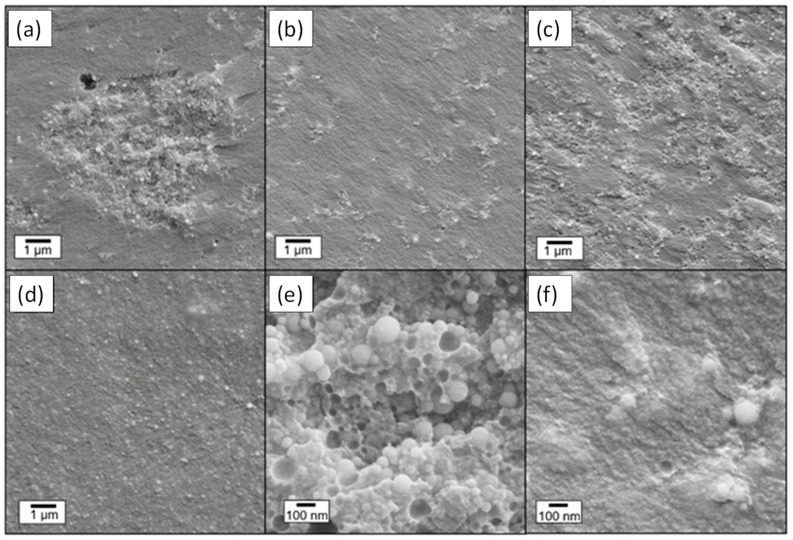
SEM images of (**a**) 10A_L, (**b**) 10AF_L, (**c**) 40A_L, (**d**) 40AF_L composites at low magnification, and (**e**) 10A_L, (**f**) 10AF_L at high magnification.

**Figure 4:**
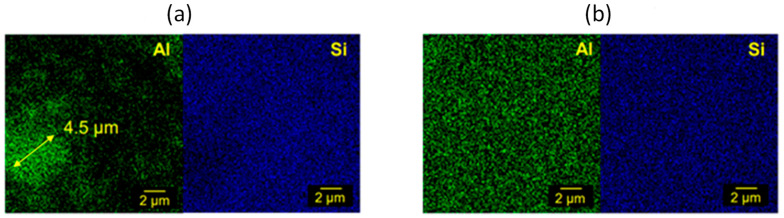
EDXS analyses on fracture surfaces of (**a**) 20A_L and (**b**) 20AF_L nanocomposites. A micrometric aggregate of alumina nanoparticles is visible in Panel (**a**).

At the SEM observation (Figure S4), the cross-section surface of the photo-cured LPMASQ sample appears dense and free of pores and macroscopic defects. Figure 3 shows SEM images of the composites’ fracture surface, indicating the presence of numerous aggregates in samples prepared with bare alumina nanoparticles (Figure 3a,c). On the other hand, cross-sectional images of samples 10AF_L and 40AF_L prepared with functionalized alumina (Figure 3b,d) reveal a much more homogeneous filler distribution even at high filler loadings (Figure 3d). Notably, this improved dispersibility of the particles is further highlighted in the higher magnification images (Figure 3e,f), warranting additional commentary. The fracture surface of sample 10A_L (Figure 3e) exhibits numerous craters caused by detached particles, suggestive of insufficient interfacial adhesion. Conversely, particles in 10AF_L are embedded in the matrix, pointing out the existence of strong interfacial interactions (Figure 3b). This is probably connected to surface functionalization, which gives the possibility to significantly increase the filler amount, preserving particle dispersion (Figure S5). The above observations are confirmed by the elemental maps obtained by energy dispersive X-ray spectroscopy (EDXS) measurements recorded on composites 20A_L and 20AF_L (Figure 4). The influence of functionalization is evident from the Al maps, which highlight the presence of micron-sized aggregates for bare particles and a homogeneous dispersion for functionalized alumina.

The thermal stability of the nanocomposites was evaluated by thermogravimetric analysis (TGA). Thermograms recorded on photo-cured LPMASQ and composites prepared with the addition of both bare (10A_L and 40A_L) and functionalized NPs (10AF_L and 40AF_L) are shown in Figure S6. Table S2 reports the decomposition temperatures T5 and T20, which correspond to the temperatures at which the sample weight loss amounts to 5% and 20%, respectively, as well as the residual weight. All samples show similar degradation curves, but the addition of aluminum oxide nanoparticles results in decreased thermal stability of silsesquioxane. Furthermore, it appears clear that alumina functionalization does not contribute to LPMASQ stability, contrary to what has been previously observed with the addition of 5 wt% of MPTMS-functionalized silica [16]. Instead, alumina functionalization leads to thermal behaviors that closely resemble those of nanocomposites with unmodified NPs, likely due to the higher amount of filler and the lower degree of grafting on the surface of alumina. Despite these findings, the advantageous impact of functionalization remains evident through the ability to incorporate extremely high ceramic filler amounts and produce LPMASQ-based samples with excellent alumina dispersion and a robust particle-matrix interface.

### 2.2. Polybutadiene/Ladder-like Polymethacryloxypropyl Silsesquioxane/Alumina Nanocomposites

#### Structural and Morphological Characterization

**Figure 5:**
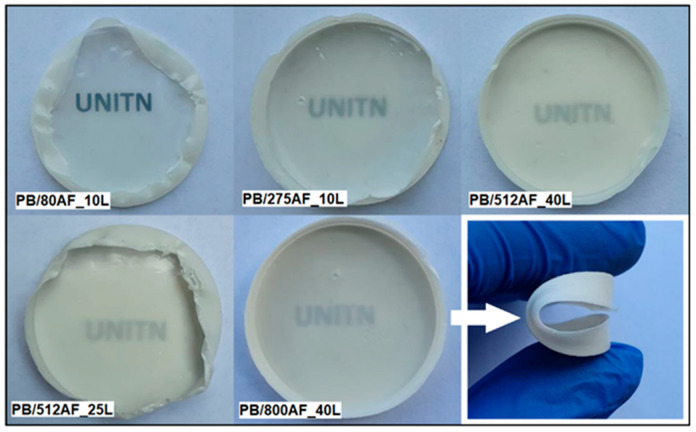
Nanocomposites produced by blending LPMASQ and PB; with increasing filler fraction, samples reduced their transparency but appeared flexible even at very high alumina loading.

Nanocomposites were also produced by incorporating functionalized alumina nanoparticles into blends of polybutadiene and LPMASQ (Section 4, Table 3, Scheme S4). Blends were produced to assess the compatibility between the components by changing the ratio between the polymer and silsesquioxane gel. The resultant samples were designated as PB/XAF_YL, where X represents the percentage of functionalized Al_2_O_3_ relative to LPMASQ, and Y denotes the weight percentage of L (LPMASQ) relative to the PB matrix. By increasing the weight percentage of LPMASQ (from 10% to 25% and 40%) in the polymer blend, it was possible to significantly increase the loading of the ceramic filler (Table 3). This resulted in the formation of homogeneous films (as seen in Figure 5) with an average thickness of 300 μm in all cases. As the filler content increases, the transparency of the samples gradually decreases, while flexibility is maintained even at very high filler fractions. It is noteworthy that the dispersal of significant amounts of alumina in the polymer blend is facilitated by the high compatibility of both functionalized particles with LPMASQ and silsesquioxane units with the organic polymer [14].

The FTIR spectra recorded on selected nanocomposites are reported in Figure S7 in comparison with the PB spectrum. It can be observed that the C=C double bond stretching vibration band is the result of the contribution of unreacted methacrylate groups (1641 cm^−1^) and polybutadiene vinyl functions (1657 cm^−1^). As the amount of ceramic particles increases, the relative intensity of the signal at 1641 cm^−1^ also increases. This is due to the contribution of the methacrylate groups on alumina particles, which is likely associated with reduced polymerization ability, as previously observed in LPMASQ/alumina composites (Table S1).

It is worth noting that the siloxane asymmetric stretching band in nanocomposites (Figure S7) shows a different lineshape compared to neat LPMASQ (Figure S1). The structural order appears modified in comparison with LPMASQ/alumina nanocomposites and pristine LPMASQ, as indicated by the modifications in the relative intensities of the two bands centered at 1020 and 1100 cm^−1^ (Figure S1) [15,17]. Specifically, the increase observed in the 1100 cm^−1^ band reflects changes in siloxane angles associated with the formation of cage-like structures resulting from chain folding, as previously reported [14]. Finally, the increased broadening of the carbonyl signal with increasing alumina loading (Figure S7) suggests the occurrence of more interactions (particularly H-bonding) taking place between particles and matrix.

Carbon-13 NMR spectra of the nanocomposites (Figure S8) show the typical resonances of pristine PB and LPMASQ without detectable shifts, confirming that the structural features of polymer backbones were not modified by photo-curing and filler addition.

**Figure 6:**
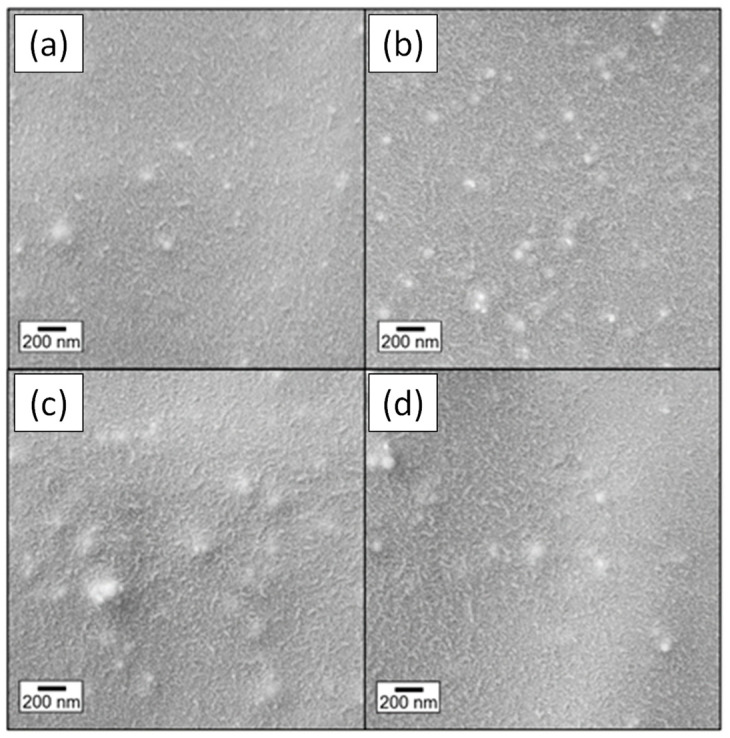
SEM images of the cryo-fracture surfaces of (**a**) PB/80AF_10L, (**b**) PB/275AF_10L, (**c**) PB/512AF_25L, and (**d**) PB/512AF_40L.

**Figure 7:**
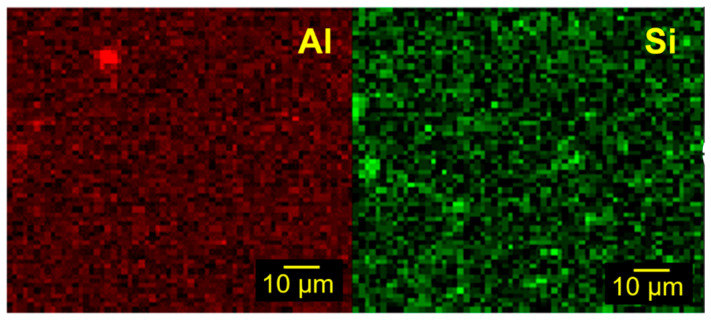
Aluminum (red) and silicon (green) EDXS elemental maps of PB/120AF_10L surface.

SEM micrographs of cryo-fractured surfaces reveal effective filler dispersion for samples made with 10% LPMASQ and varying alumina loads (Figure 6a,b). The pictures depict well-distributed particles within the polymeric matrix with little aggregation, highlighting robust interactions between the two. Increasing LPMASQ content up to 25 and 40% (Figure 6c,d) brings about a minor increase in fracture surface roughness but still ensures acceptable particle dispersal in high loadings. These observations are supported by Al and Si EDXS elemental maps acquired on the PB/120AF_10L surface (Figure 7). As indicated in the Si map, LPMASQ is distributed evenly throughout PB, although areas with micrometer-sized LPMASQ accumulation are also visible, which is consistent with previous studies [14]. Even though a large aggregate (~5 μm) can be observed, functionalized alumina nanoparticles are evenly distributed across the entire analyzed region, revealing a valuable chemical affinity between the particles and matrix. The dispersion ability of the LPMASQ phase in the PB matrix probably governs the spreading of nanoparticles in the composite, also considering the interactions pointed out by the infrared analysis (Figure S7). The significance of achieving a uniform distribution in the composite is pertinent to properties related to its structure, such as mechanical and thermal properties, as well as the potential for creating highly transparent films (Figure 5).

**Figure 8:**
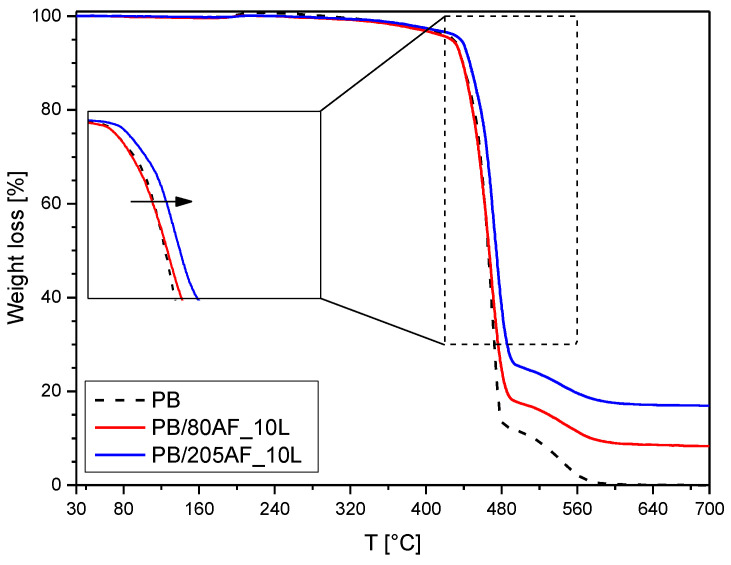
Thermogravimetric analyses of the PB-based composites; the magnification of the range from 420 to 560 °C is shown in the inset.

The thermal stability of PB-based nanocomposites was evaluated by TGA. Figure 8 shows the thermograms recorded on neat PB and representative samples prepared with 10 wt% of LPMASQ and two different alumina loadings. The TGA curve of the PB sample is characterized by two decomposition steps. According to McCreedy et al. [62], the main mass loss step (endset temperature 480 °C, mass loss 87%) is attributed to depolymerization reactions by chain scission, with butadiene evolution. The large amount of weight loss in the first step suggests that the degree of crosslinking of PB is low, as can be expected in the case of photo-curing [62]. The second mass loss step (from 480 to 590 °C, 13%) is due to the degradation of highly crosslinked and cyclized PB residues [62].

The shapes of the TGA thermograms of nanocomposites are similar to the one of neat PB, but the endset temperature of the first degradation step increases (486 and 495 °C for PB/80AF_L and PB/205AF_L, respectively), suggesting a slight increase in network crosslinking; the residual masses also increase, due to the inorganic components. To better evaluate the thermal stability of the samples, the calculated values of T5 and T20 and residual masses are reported in Table S2.

At a lower filler fraction (sample PB/80AF_10L, 1.7 vol%), the alumina seems to have little effect on the thermal stability of polybutadiene, as the onset temperature of thermal degradation is substantially unchanged. With increasing filler fraction (sample PB/205AF_10L, 5.1 vol%), the thermal stability slightly increases, as can be observed from the inset of Figure 8. However, increasing the concentration of LPMASQ up to 40 wt% reduces the onset of thermal degradation, which further progressively decreases as the filler loading increases (Table S2), probably due to the observed negative effect of alumina on the thermal stability of LPMASQ (Figure S6).

### 2.3. Thermal Conductivity of Nanocomposites

LFA Measurements

The thermal conductivity (TC) properties of LPMASQ/alumina nanocomposites were investigated to study the effect of alumina loadings and particle functionalization. Furthermore, since PB is a largely available and low-cost polymer widely employed in electronic packaging, we explored the thermal conductivity of blends produced with the lowest LPMASQ content (10 wt%) and variable alumina loadings, which exhibited good thermal stability (Figure 8). The TC of PB composites containing higher (25 and 40 wt%) LPMASQ amounts were also evaluated.

Composites thermal conductivity was calculated according to Equation (1):

(1) $$k=\rho \cdot c_{P}\cdot \alpha \;$$

where ρ is the density (g cm^−3^), $c_{P}$ the specific heat capacity at constant pressure (J K^−1^ g^−1^), and α the thermal diffusivity (mm^2^ s^−1^). The obtained TC values are reported in Table 1 and Table S3, along with the values of ρ, $c_{P}$, and α.

**Table 1:** Calculated values of thermal conductivity, according to Equation (1), for all of the produced composites. The percentage increase (gain) over the respective matrices (L or PB_YL with Y = 10, 25, 40) is also reported.

Sample	TC (W m^−1^ K^−1^)	TC Gain (%)	Sample	TC (W m^−1^ K^−1^)	TC Gain (%)
L	0.68 ± 0.05	-	PB	0.18 ± 0.01	-
10A_L	0.72 ± 0.05	+5.9%	PB_10L	0.19 ± 0.01	+5.5%
20A_L	0.85 ± 0.05	+25%	PB/80AF_10L	0.22 ± 0.01	+15.8%
40A_L	1.11 ± 0.06	+63.2%	PB/120AF_10L	0.25 ± 0.01	+31.6%
10AF_L	0.70 ± 0.05	+2.9%	PB/205AF_10L	0.28 ± 0.01	+47.4%
20AF_L	0.79 ± 0.05	+16.2%	PB_25L	0.21 ± 0.01	+16.6%
40AF_L	0.98 ± 0.06	+44.1%	PB/512AF_25L	0.42 ± 0.01	+100.0%
80AF_L	1.22 ± 0.06	+79.4%	PB_40L	0.22 ± 0.01	+22.2%
120AF_L	1.38 ± 0.06	+102.9%	PB/512AF_40L	0.39 ± 0.01	+77.3%
-	-	-	PB/800AF_40L	0.47 ± 0.01	+113.6%

Photo-crosslinked LPMASQ (sample L) shows a remarkable TC value of 0.68 W m^−1^ K^−1^, significantly higher than typical unfilled silicone rubber (0.16 W m^−1^ K^−1^ for PDMS) and the highly crosslinked silicone resins developed by Jie et al. [63] (0.21 W m^−1^ K^−1^). The high TC value of LPMASQ might be attributed to its structure, characterized by high bond density due to both fully condensed trifunctional units in ladder-like structures and polymerized organic side chains. Moreover, weak interactions, such as those that probably occur in crosslinked LPMASQ, leading to ladders chain-folding, are reported to strongly contribute to the thermal conductivity of the polymers [55]. The introduction of alumina nanoparticles results in an increase in TC, albeit without any significant difference between the bare and functionalized particles. However, composites of LPMASQ with high alumina loadings were only achievable through the use of functionalized nanoparticles, as Section 2.1 details. This led to noteworthy thermal conductivity values for samples 80AF_L and 120AF_L, with the latter showing around a 103% increase compared to L. It should be noted that the grafting density of the modified alumina may be insufficient (1.7 wt%) to significantly affect the interface chemistry and induce TC differences. However, surface modification enabled the increase in filler loading without any adverse effects on filler dispersion.

Blending PB with LPMASQ resulted in a slight increase in thermal conductivity (TC) in comparison to PB. The TC increased by approximately 17% and 22% for the composite with 25 wt% and 40 wt% of LPMASQ, respectively, in the absence of alumina. The addition of alumina to PB_10L produced a net increase in TC up to ca. 47% compared to the matrix. Increasing the alumina content resulted in higher TC values in the composites containing 25 and 40 wt% of LPMASQ, up to ca. 100% and 114% compared to the matrix. It is worth mentioning that measurements have been performed on small specimens extracted from different regions of the prepared materials. Moreover, small and undesirable changes in the shape and weight of the samples could potentially affect the precision of experimental values. Furthermore, local inconsistencies such as nanoporosity, inhomogeneity, or defectivity cannot be ruled out completely but can be limited through improved processing procedures. Nevertheless, the thermal conductivity values demonstrate a clear trend confirming the enhancement of the thermal transport capacity of nanocomposites.

To gain insights into the correlation between thermal behavior and the microstructure of the nanocomposites, we compared the experimental results with three analytical models described in the experimental part, i.e., the linear mixture rule (parallel model), the inverse mixture rule (series model), and the Maxwell–Garnett model. These models have been previously used in the literature to predict the thermal properties of nanocomposites based on different microstructural assumptions. We applied the three aforementioned models using both (i) the thermal conductivity of bulk γ-Al_2_O_3_ (35 W m^−1^ K^−1^) [64], following a procedure often reported in the literature, and (ii) the TC values calculated from the experimental values of density, thermal diffusivity, and specific heat of bare alumina NPs (3.95 g cm^−3^, 0.247 mm^2^ s^−1^, and 4.65 J g^−1^ K^−1^, respectively) and modified alumina NPs (3.95 g cm^−3^, 0.37 mm^2^ s^−1^, and 2.72 J g^−1^ K^−1^, respectively), which take into account the nanostructured nature of the filler.

**Figure 9:**
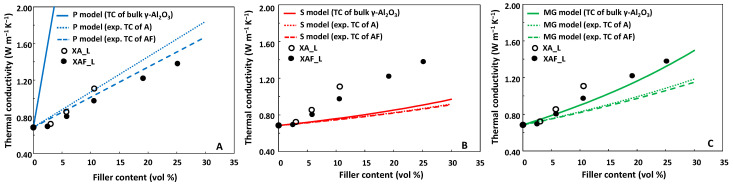
Thermal conductivity values of LPMASQ nanocomposites containing bare (A, empty circles) and surface modified (AF, black circles) alumina as a function of the filler’s volumetric fraction. Curves in panels (**A**–**C**) are the TC values calculated according to the parallel (P, blue lines), series (S, red lines), and the Maxwell–Garnett (MG, green lines) models, respectively. Solid lines have been obtained using the TC values of bulk γ-Al_2_O_3_ and for the dotted and dashed lines by using experimental TC values of bare and surface-modified alumina, respectively.

**Figure 10:**
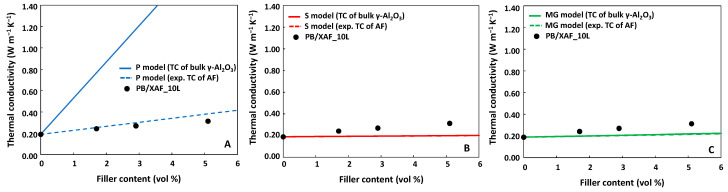
Thermal conductivity values of PB/XAF_10L nanocomposites as a function of the filler’s volumetric fraction (black circles). Curves in panels (**A**–**C**) are the TC values calculated according to the parallel (P, blue lines), series (S, red lines), and the Maxwell–Garnett (MG, green lines) models, respectively. Solid lines have been obtained by using the TC values of bulk γ-Al_2_O_3_ and for the dashed lines by using experimental TC values of the surface-modified alumina.

Results are shown in Figure 9 for the LPMASQ nanocomposites and Figure 10 for the PB/LPMASQ ones.

When the models are applied by considering the TC value of bulk γ-Al_2_O_3_ (solid lines), the Maxwell–Garnett (MG) model (Figure 9C) is the only one that accurately approximates the experimental results. On the other hand, the parallel and the series models (Figure 9A,B) respectively overestimate and underestimate the retrieved TC values, as frequently reported in the literature [49]. The hypotheses underlying the MG model consider (i) the existence of spherical filler particles that (ii) do not interact with each other, and (iii) maintain complete contact with the matrix, (iv) without any interfacial thermal resistance (ITR). The features of the nanocomposites, as highlighted through the structural and morphological characterization, fit at least the first three assumptions. Surface functionalization facilitates the dispersion of filler nanoparticles, which become embedded in the matrix and do not undergo significant interactions. To confirm the negligible impact of interparticle interactions, as specified by the fourth assumption of the MG model, we conducted a best-fitting analysis of experimental data utilizing the Tavangar model (outlined in the experimental section) with ITR parameter manipulation. The ITR values obtained in this way are 1.5∙10^−8^ ± 3∙10^−9^ and 1.45∙10^−8^ ± 6∙10^−10^ m^2^ K W^−1^ for the samples containing bare and modified alumina, respectively. These small values further validate the satisfactory fitting of the experimental results using the MG model. It is worth noting that the condition of ITR = 0 is an assumption of all three models.

When the models are applied using the experimental TC values of the filler, no substantial changes can be noticed in the case of the series model, while the parallel model applied satisfactorily fits the experimental TC values of the nanocomposites both for the bare (dotted red line) and modified (dashed red line) alumina. This can be explained by noting that the parallel model does not take into account the particle-like nature of the filler and provides a weighted average of the thermal conductivity contributions of the constituents without considering the filler features. This aspect is mitigated by using the experimentally determined TC values of the nanostructured filler. On the other hand, since this information is already intrinsically contained in the MG model, this model fails to adequately match the experimental thermal conductivity values of the nanocomposites (dashed and dotted green lines in Figure 9C).

Similar considerations apply also to the PB/XAF_10L nanocomposites. The dependence of the TC on the filler content and the results of the modeling by the parallel, series, and MG approaches are shown in Figure 10.

Moreover, in this case, the parallel model with the experimentally measured TC values of the filler satisfactorily fits the experimental values (blue dashed line in Figure 10A). Notably, the Tavangar model accepts an ITR value of 1.04∙10^−7^ ± 1∙10^−10^ for PB nanocomposites, which is one order of magnitude higher than the corresponding values obtained for the LPMASQ-based nanocomposites. The higher ITR value obtained when PB is used with LPMASQ implies that heat transfer within the polymeric blend is more difficult than when the filler is incorporated into the LPMASQ alone. Moreover, the PB/LPMASQ blends did not show a significant TC increase with increasing the amount of LPMASQ (Figure S9) in the absence of filler, notwithstanding the higher TC of LPMASQ (0.68 W m^−1^ K^−1^) compared to PB (0.18 W m^−1^ K^−1^). This suggests that while the inclusion of LPMASQ enhances the mechanical and thermal resistance, as previously shown [18], the affinity between the two polymeric phases with regard to heat transfer is weak. In other words, an increase in the LPMASQ content hinders the potential benefits of adding a more conductive component due to interfacial limitations.

On the other hand, the real advantage consists in the possibility of using LPMASQ as a “filler carrier,” i.e., a component capable of increasing the filler loading (and possibly the TC) further due to the affinity between LPMASQ and the functionalized filler, without impacting the flexibility and homogeneity of the nanocomposite (Figure 5).

**Figure 11:**
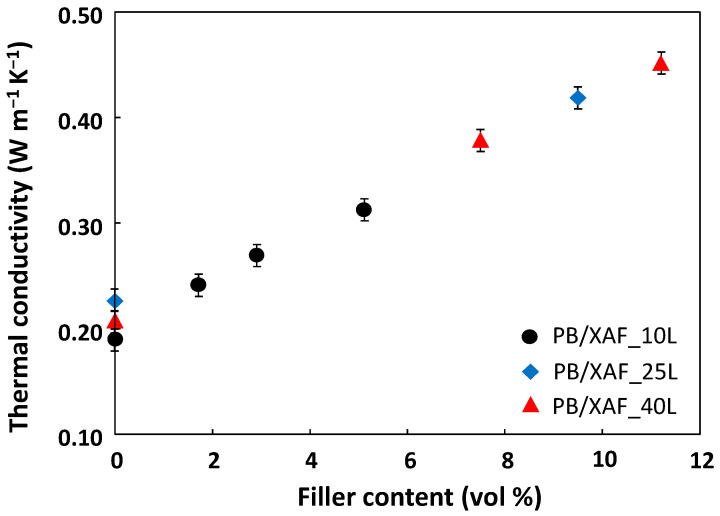
Thermal conductivity values of PB/XAF_L nanocomposites as a function of the filler’s volumetric fraction at different amounts of LPMASQ, i.e., 10 wt% (black circles), 25 wt% (blue rhombi), and 40 wt% (red triangles).

Bearing this in mind, we measured the TC values at various filler contents for nanocomposites containing 10 wt%, 25 wt%, and 40 wt% of LPMASQ. The results (Table 1) are shown in Figure 11.

Increasing the amount of LPMASQ from 10 to 40 wt% enables the preparation of nanocomposites with a filler loading exceeding two times higher (11.2 vol%) compared to the maximum filler amount (5.1 vol%) introducible in the composites containing 10 wt% LPMASQ without adversely impacting viscosity, flexibility, and filler dispersion. Accordingly, a notable increase in the thermal conductivity (TC) of 113.6% in comparison to the matrix was achieved with 40 wt% LPMASQ, whereas only 47.4% was reached with 10 wt% LPMASQ.

This result is relevant from an applicative point of view due to the remarkable increase in TC of flexible nanocomposites with relatively low filler content when compared with the literature results [65,66]. Furthermore, it allows a better unveiling of the role of LPMASQ for thermal management applications in comparison with the cage-like analog POSS, which has been recently reported [44]. The enhancement in TC in the presence of POSS within the nanocomposite was of ca. 60–70% at filler contents of 10–15 vol%. In fact, the functionalized alumina nanoparticles were decorated with POSS by a separate synthetic step before being embedded into the PB matrix. The highly reactive termination of POSS nano-units ensured compatibility between the filler and matrix, as well as interactions between the fillers. These factors, in conjunction with the configuration of POSS units, have a direct impact on the TC of the resulting nanocomposites.

On the other hand, the role of the silsesquioxane unit in the present study is very different since LPMASQ is a component of the blend together with PB rather than a filler decorating moiety. In the present study, the thermal conductivity increased by up to 113.6% at comparable filler loadings. This exceptional outcome was achieved by merely combining PB, LPMASQ, and MPTMS-functionalized alumina components in a one-pot process. Due to its intrinsic structural features and affinity with the functionalized alumina, LPMASQ units result in greater increases in thermal conductivity compared to POSS. This occurs indirectly as LPMASQs primarily serve as efficient filler carriers, facilitating the preparation of flexible nanocomposites with higher filler loadings.

## 3. Conclusions

The study showcases, for the first time, the effectiveness of ladder-like poly(methacryloxypropyl) silsesquioxane gels as a precursor to high thermal conductivity nanocomposite polymers. By adding varying amounts of a ceramic filler (γ-Al_2_O_3_), bare or functionalized, into the LPMASQ matrix, the thermal conductivity (TC) of the resulting nanocomposites increased up to approximately 103% compared to the LPMASQ matrix. Notably, functionalizing the filler’s surface with the same moiety as the organic pendants of LPMASQ (methacryloxypropyl chains) improved the filler dispersion and enhanced the maximum filler loading from 10.6 to 25.1 vol% while avoiding the introduction of macroscopic defects.

Blends of LPMASQ with polybutadiene have also been used as the matrix in order to confer higher flexibility to the nanocomposites, still keeping the desired microstructural properties. It has been demonstrated that in this system, LPMASQ mainly acts as a “filler carrier” due to its chemical affinity with the surface of the modified ceramic nanoparticles. Increasing the amount of LPMASQ in the nanocomposites up to 40 wt% allows for the dispersion of a higher amount of filler. This results in nanocomposites with enhanced thermal conductivity, up to 113.6% higher compared to the matrix. Analytical models have been used to elucidate the correlation between thermal behavior and microstructural features of nanocomposites. The study has demonstrated that the parallel model applied, considering the particle-like characteristics of the filler, aligns well with the experimental TC values of the nanocomposites. This indicates that the assumptions of the model, i.e., negligible interfacial thermal resistance (ITR), non-interacting, and homogeneously distributed filler, are key features of the nanocomposites and are consistent with the results of morphological and structural analyses. The ITR determined through a best-fit analysis of experimental TC values is one order of magnitude greater in nanocomposites containing both PB and LPMASQ compared to those with LPMASQ alone. This indicates that the heat transfer is more difficult through the polymeric blend. For this reason, to further improve the thermal conductivity performance of blends, it is desirable to selectively tune the chemical interface between the polymeric phases by modifying the organic functionalities of ladder-like silsesquioxanes.

## 4. Materials and Methods

### 4.1. Materials

Methacryloxypropyl trimethoxysilane (MPTMS, 98%) was purchased from ABCR GmbH (Karlsruhe, Germany). γ-Alumina nanoparticles (NanoArcTM, AL-0405, 99.5%, specific surface area 35 m^2^ g^−1^, average particle size 48 nm) were purchased from Alfa Aesar (Haverhill, MA, USA). Polybutadiene (PB cis, average MW 200,000–300,000), potassium carbonate (≥99.0%), toluene (99.8%), tetrahydrofuran (THF, 99.9%), and 1-hydroxycyclohexyl phenyl ketone (Irgacure 184, 99%), were obtained from Sigma-Aldrich (St. Louis, MO, USA).

### 4.2. Preparation of Samples

LPMASQ Synthesis. Ladder-like poly(methacryloxypropyl)silsesquioxane was synthesized according to an already published procedure [14,16,17,61]. Briefly, K_2_CO_3_ (0.04 g) was dissolved in a mixture of deionized H_2_O (4.8 mL) and THF (9 mL). MPTMS (0.08 mol) was added dropwise under nitrogen flow, and the clear solution was stirred at RT for 240 h in the dark. A transparent viscous product was obtained by solvent evaporation under reduced pressure at 30 °C (yield of 95%), which was stored in the dark under a nitrogen atmosphere. The described procedure is briefly reported in Scheme S1.

Nano-Al_2_O_3_ Functionalization. 2 g of γ-Al_2_O_3_ were dispersed in 50 mL of toluene by sonication (15 min). Then, MPTMS (864.2 mg, 827 µL) was added dropwise in a 2:1 molar ratio compared to the surface -OH groups of alumina particles (0.84 mmol g^−1^) [44], and the mixture was refluxed at 120 °C under vigorous stirring for 24 h. The particles, labeled MPTMS@Al_2_O_3_, were recovered by centrifugation at 4500 rpm for 15 min, washed three times with toluene, and allowed to dry in vacuum at 80 °C overnight. The described procedure is briefly reported in Scheme S2.

Preparation of LPMASQ/Al_2_O_3_ nanocomposites. LPMASQ (1 g) was dissolved in 1 mL of THF at room temperature. Suspensions of bare and functionalized Al_2_O_3_ nanoparticles in THF were prepared by sonication (15 min) and added to the LPMASQ solution under stirring; then, 10 mg of Irgacure 184 photo initiator dissolved in THF was added, and the mixture was stirred for 30 min, avoiding exposure to light. The obtained final suspension was drop-casted onto square Teflon^®^ substrates and dried overnight in the dark. Then, 100, 200, and 400 mg of bare and functionalized alumina NPs were respectively used to produce composites at 10, 20, and 40 wt% of powders with respect to the LPMASQ matrix; a sample was also prepared without filler particles in the same conditions. Samples were placed on a tray chilled by melting ice in an isolated chamber under nitrogen flow and photo-crosslinked using a mercury vapor lamp (OSRAM, HBO 50 W/AC 39 V, lamp-specimen distance 12 cm, curing time 10 min). The described procedure is briefly reported in Scheme S3. It is worth noting that it was possible to prepare nanocomposites with higher alumina amounts (80 and 120 wt%) only using functionalized NPs. These compositions were crosslinked in glass Petri dishes by applying 10 min irradiation to the top side of the sample followed by 5 min irradiation of the bottom side in order to improve sample photo-curing. The obtained composites were stored under a N_2_ atmosphere in the dark. Samples are labeled as XA(or AF)_L, where X is the weight fraction of A (bare alumina) and AF (functionalized alumina) with respect to L, which indicates the LPMASQ matrix. The composition of the nanocomposites in terms of volume fractions, listed in Table 2, has been calculated starting from the weight fractions and density values experimentally measured (Table S3), according to Equations (S2)–(S4).

**Table 2:** Sample labeling and alumina volume fraction of the produced LPMASQ/alumina nanocomposites.

Sample	Al_2_O_3_ Volume Fraction (vol%)
L	0.0
10A_L	3.0
20A_L	5.7
40A_L	10.6
10AF_L	2.4
20AF_L	5.8
40AF_L	10.5
80AF_L	19.1
120AF_L	25.1

Preparation of PB/LPMASQ/alumina nanocomposites. Nanocomposites made of PB, 10, 25, and 40 wt% of LPMASQ with respect to PB and different loadings of functionalized alumina were prepared through a similar procedure. LPMASQ-alumina suspensions were prepared as described above and added to a solution of PB (1 g) in THF (10 mL). After the addition of Irgacure 184 solution (10 mg, THF 0.5 mL), the mixture was vigorously stirred for 24 h in the dark [14], then poured in PTFE circular molds (ϕ = 4 cm) and degassed at room temperature in a vacuum with two fast cycles (~2 min each). The samples were kept drying overnight under the hood at room temperature in the dark. Finally, the composite films were photo-crosslinked in the same conditions used for LPMASQ composites. The obtained films were stored in an N_2_ atmosphere in the dark. The described procedure is briefly reported in Scheme S4. Samples were labeled as PB/XAF_YL, where X is the percentage of functionalized Al_2_O_3_ with respect to LPMASQ and Y is the weight percentage of L (LPMASQ) with respect to the PB matrix. Labels and compositions are reported in Table 3.

**Table 3:** Sample labeling, LPMASQ weight percentage, and alumina volume fraction of the produced PB/LPMASQ/alumina nanocomposites.

Sample	LPMASQ (wt%)	Al_2_O_3_ Volume Fraction (vol%)
PB	0.0	0.0
PB_10L	10.0	0.0
PB/80AF_10L	10.0	1.7
PB/120AF_10L	10.0	2.9
PB/205AF_10L	10.0	5.1
PB_25L	25.0	0.0
PB/512AF_25L	25.0	9.5
PB_40L	40.0	0.0
PB/512AF_40L	40.0	7.5
PB/800AF_40L	40.0	11.2

### 4.3. Characterization Techniques

The functionalization of alumina particles with MPTMS was studied using Fourier transform infrared (FTIR) spectroscopy. FTIR spectra of bare and modified alumina were recorded on KBr pellets with a Nicolet Avatar 330 spectrometer in transmittance mode in the 4000–400 cm^−1^ wavenumber range (64 scans, resolution 4 cm^−1^). Structural information on nanocomposites samples was obtained by recording the attenuated total reflectance (ATR) FTIR spectra, which were collected with a Varian 4100 Excalibur spectrometer equipped with a diamond ATR crystal (4000–550 cm^−1^, 64 scans, resolution 4 cm^−1^).

Both particle functionalization and the structural features of nanocomposites were characterized by magic angle spinning nuclear magnetic resonance (MAS NMR) analyses that were performed using a Bruker 400 WB spectrometer (Bruker, Billerica, MA, USA) operating at a proton frequency of 400.13 MHz. NMR spectra were acquired with single pulse and cross-polarization (CP) pulse sequences under the following conditions: ^13^C frequency: 100.48 MHz, contact time 2 ms, decoupling length 5.9 µs, recycle delay: 5 s, 2 k scans. ^29^Si frequency: 79.48 MHz, contact time 5 ms, decoupling length 6.3 µs, recycle delay: 10 s, 2 k scans. Single pulse sequence: π/4 pulse 3.9 µs, recycle delay 300 s, 1 k scans. Samples were packed in 4 mm zirconia rotors, which were spun at 8 kHz under airflow. Adamantane and Q_8_M_8_ were used as the external secondary references.

To quantify the degree of particles’ functionalization [67], TGA curves of bare and modified alumina powders were acquired by a Q5000 TA Instruments thermogravimetric analyzer under constant N_2_ flow (10.0 mL/min) from 30 °C to 700 °C at a heating rate of 10 °C/min. TGA analyses of the photo-cured nanocomposites were acquired with a Mettler Toledo TG50 in constant airflow (15 mL/min) from 30 °C to 700 °C with a heating rate of 10 °C/min.

Images of the fracture surfaces of composites were obtained with a Carl Zeiss Gemini Supra 40 field emission SEM operated at 7.50 keV (Carl Zeiss, Oberkochen, Germany), using secondary electrons as the main signal. EDXS maps were obtained using a Jeol JSM 5500 scanning electron microscope (SEM) operating at 20 kV equipped with an energy-dispersive X-ray (EDX) analyzer (working distance of 20 mm).

Density values of the Al_2_O_3_ powders, LPMASQ/Al_2_O_3,_ and PB/LPMASQ/Al_2_O_3_ nanocomposites were measured using a Micrometrics Multivolume Helium Pycnometer Accupyc 1330 (Micrometrics, Norcross, GA, USA), at room temperature.

Specific heat capacity values of crosslinked LPMASQ and bare and modified alumina were evaluated using a Mettler Toledo 30 DSC equipped with a low cell temperature cell, according to the ASTM E1269 standard, at 25 °C. As-obtained specific heat values were used to extrapolate the specific heat of LPMASQ nanocomposites using the mixture rule reported in Equation (2)

(2) $$c_{P}{}_{c}=c_{P}{}_{m}\cdot w_{m}+c_{P}{}_{f}\cdot w_{f}$$

where *c_Pc_*, *c_Pm_*, and *c_Pf_* are the composite, matrix, and filler-specific heat capacities, respectively; *w_m_* and *w_f_* are the matrix and filler weight fractions, respectively [68]. The specific heat of the PB was evaluated alongside thermal diffusivity using a Netzsch LFA 467 HyperFlash Light Flash Analyzer; measurements were run with a laser voltage of 250 V, pulse width of 1000 ms, five pulses per sample, under N_2_ flow at 25 °C. The specific heat of PB nanocomposites was calculated according to Equation (2) by considering the matrix as a mixture of PB and LPMASQ. Modified and bare Al_2_O_3_ nanoparticles thermal diffusivity was analyzed using the same LFA analyzer in a three-layer setup, pressed between two aluminum discs (clamping torque of 15 cN m) and measured with a laser voltage of 250 V, pulse width of 0.60 ms, five pulses per sample, under N_2_ flow at 25 °C. Thermal diffusivity of nanocomposites was measured for irregularly shaped thin films with the same measurement parameters used for the analysis of powders.

### 4.4. Thermal Conductivity Modelling

The experimental values of the thermal conductivity of the prepared composites were qualitatively compared with the theoretical conductivity calculated via some models from the literature [49,69] to obtain a structural description of the composites and clarification of the interface properties between the different phases. The models used, in which k_c_, k_m_, and k_f_ are the composite, matrix, and filler thermal conductivity, respectively, and ϕ is the filler volume fraction, are listed below:

- Linear mixture rule (parallel model) [49]: Equation (3). This model assumes uniform temperature gradient and heat flux as the weighted sum of heat fluxes through matrix and filler regions. The filler and matrix independently contribute to the thermal conductivity according to their relative volume fractions. The model is implicitly based on the assumption of perfect contact between particles.
  (3) $$k_{c}=\left(1-\phi \right)k_{m}+\phi k_{f}\;$$
- Inverse mixture rule (series model) [49]: Equation (4). This model assumes uniform heat flux and temperature gradient as the weighted sum of temperature gradients through matrix and filler regions. The series model is most applicable for fillers dispersed in a matrix without percolation, even at high filler loadings.
  (4) $$k_{c}={\left(\frac{\left(1-\phi \right)}{k_{m}}+\frac{\phi }{k_{f}}\right)}^{-1}$$
- Maxwell–Garnett model [49,70]: Equation (5), where *δ* = *k_f_*/*k_m_*. The model represents an exact solution for the effective medium approximation (EMA) for non-interacting, homogeneously distributed spherical particles.
  (5) $$k_{c}=k_{m}\left(1+\frac{3\phi \left(\delta -1\right)}{2+\delta -\phi \left(\delta -1\right)}\right)$$

These models have been applied by considering both the TC values of the filler experimentally obtained as mentioned above and the TC value of bulk γ-Al_2_O_3_ reported in the literature (35 W m^−1^ k^−1^) [64].

The model developed by Tavangar et al. [71] has been used to estimate the average interfacial thermal resistance (ITR) of the produced nanocomposites. Its expression is reported by Equation (6) [69], where a is the spherical particle radius. The simulation was run using the OriginPro 2018 Software.

(6) $$1-\phi =\frac{k_{m}^{\frac{1}{3}}\cdot \left(k_{f}k_{c}ITR+ak_{c}-ak_{f}\right)}{k_{c}^{\frac{1}{3}}\cdot \left(k_{f}k_{m}ITR+ak_{m}-ak_{f}\right)}$$

## Data Availability

The data presented in this study are available on request from the corresponding author.

## Supplementary Materials

The following supporting information can be downloaded at https://www.mdpi.com/article/10.3390/gels9100810/s1, Scheme S1: Synthesis of LPMASQ; Scheme S2: Nano-alumina functionalization; Scheme S3: Preparation of LPMASQ/Al_2_O_3_ nanocomposites; Scheme S4: Preparation of PB/LPMASQ/alumina nanocomposites; Figure S1: ATR-FTIR spectrum of as-synthetized LPMASQ; Figure S2: TG analyses of bare (black) and functionalized (red) alumina nanoparticles; Figure S3: ^29^Si CPMAS NMR spectrum of photo-cured L sample; Figure S4: SEM cross-section image of L sample at high and low magnification; Figure S5: SEM cross-section image of sample 80AF_L; Figure S6: TGA curves of photo-cured LPMASQ (blue) and LPMASQ/alumina nanocomposites with both bare (black) and functionalized NPs (red); Figure S7: ATR-FTIR spectra of PB/LPMASQ/AF nanocomposites superimposed to the spectrum of PB; the signals of LPMASQ and modified nanoparticles are highlighted. On the right: magnifications of both the siloxane band in the 1200–950 cm^−1^ range and the C=O and C=C stretching vibrations region; Figure S8: ^13^C CPMAS NMR spectrum of PB/120_AF_10L nanocomposite, as a representative sample; Figure S9: Thermal conductivity values for nanocomposites containing PB and different amounts of LPMASQ; Table S1: Degree of polymerization (DP) calculated from the integration of d and d’ resonances in ^13^C CPMAS NMR spectra of LPMASQ/alumina nanocomposites; Table S2: Results of the TG analyses of LPMASQ/alumina and PB/LPMASQ/alumina nanocomposites: decomposition temperatures T5 and T20 correspond to the temperature at which the sample weight losses are 5% and 20%, respectively; the residual mass is calculated at 700 °C; Table S3: Density, specific heat, thermal diffusivity, and thermal conductivity values of the prepared samples.
