# Supplementary material for: Ladder-like Poly(methacryloxypropyl) silsesquioxane-Al2O3-polybutadiene Flexible Nanocomposites with High Thermal Conductivity
Source: Gels. 2023 Oct 10;9(10):810. doi: 10.3390/gels9100810 (PMC10606264; doi:10.3390/gels9100810)
Supplement: Supplementary file 1 [file gels-09-00810-s001.zip › gels-2624761-supplementary.pdf]

Supplementary Materials

Ladder-like poly(methacryloxypropyl) silsesquioxane-Al_2O_3-polybutadiene flexible nanocomposites with high thermal conductivity

Pietro Mingarelli [1], Chiara Romeo [1], Emanuela Callone [1,2], Giulia Fredi [1], Andrea Dorigato [1], Massimiliano D'Arienzo [3], Francesco Parrino [1*], Sandra Dirè [1,2*]

[1] University of Trento, Department of Industrial Engineering, via Sommarive 9, 38123 Trento, Italy; petrumingarelli@gmail.com (PM); chiara.romeo@unitn.it (CR); emanuela.callone@unitn.it (EC); giulia.fredi@unitn.it (GF); andrea.dorigato@unitn.it (AD); francesco.parrino@unitn.it (FP); sandra.dire@unitn.it (SD)
[2] University of Trento, "Klaus Müller" Magnetic Resonance Lab., via Sommarive 9, 38123 Trento, Italy; emanuela.callone@unitn.it (EC); sandra.dire@unitn.it (SD)
[3] University of Milano-Bicocca, Department of Materials Science, INSTM, via R. Cozzi 55, 20125 Milano, Italy; massimiliano.darienzo@unimib.it
* Correspondence: sandra.dire@unitn.it (SD); francesco.parrino@unitn.it (FP)

Preparation of samples

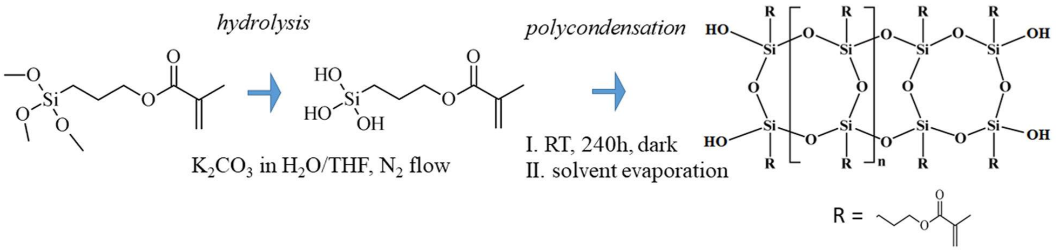

Scheme S1. Synthesis of LPMASQ.

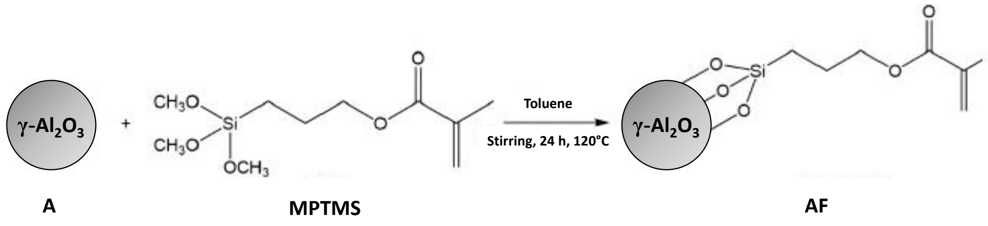

Scheme S2. Nano-alumina functionalization.

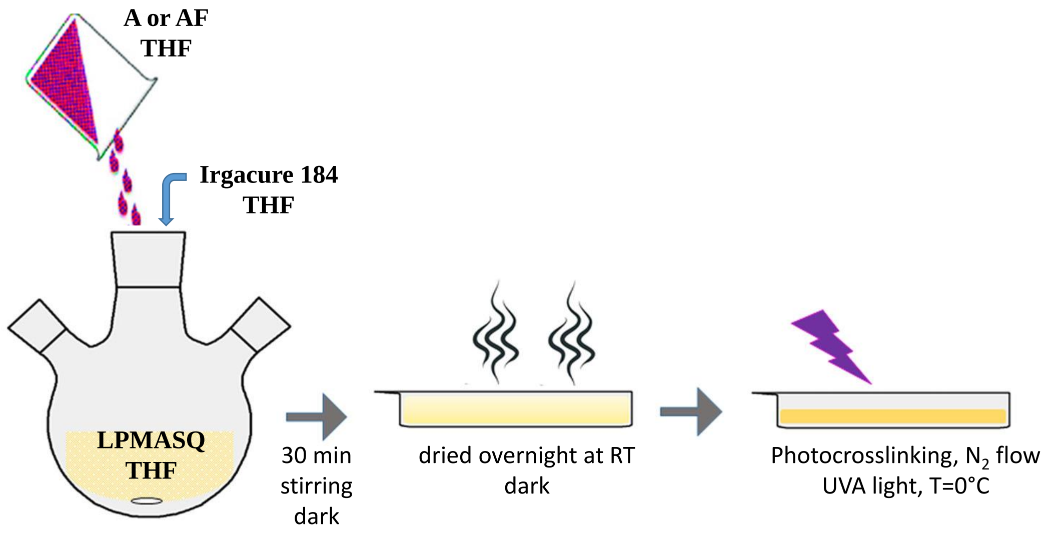

Scheme S3. Preparation of LPMASQ/Al_2O_3 nanocomposites.

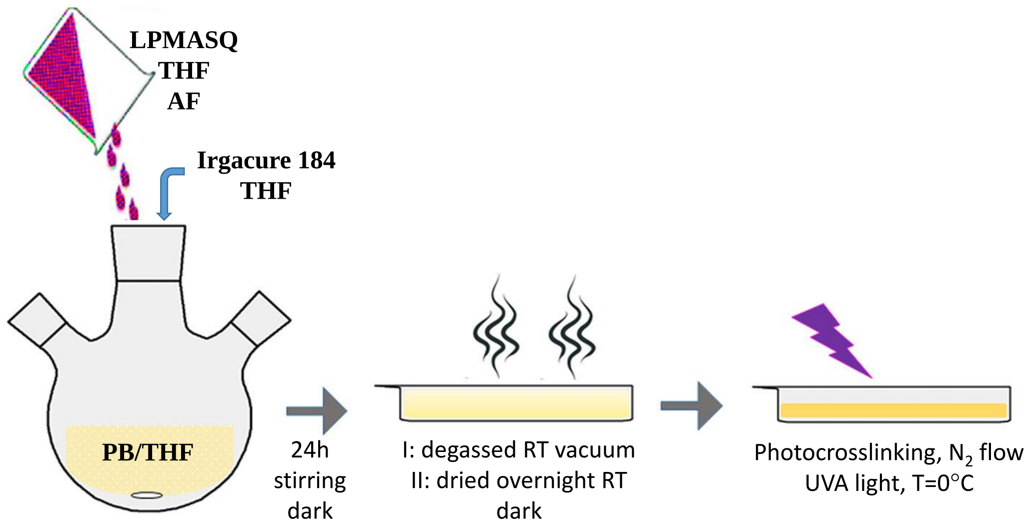

Scheme S4. Preparation of PB/LPMASQ/alumina nanocomposites.

LPMASQ characterization

The ATR-FTIR spectrum of LPMASQ gel (Figure S1a) assesses the reproducibility of the sol-gel synthesis. Methacrylate C=O and C=C stretching vibrations are detected at 1712 and 1637 cm^{-1}, respectively. The inset shows the magnification of the region between 1200 and 900 cm^{-1} to highlight the complex band associated to Si-O asymmetric stretching vibrations, which is characterized by two main bands centred at 1103 and 1020 cm^{-1} respectively, proving the ladder-like structure of prepared silsesquioxanes. The bands at 1103 and 1163 cm^{-1} can be attributed respectively to cage-like structures that are probably formed by silsesquioxane-ladder chain folding and poly-cages; the lower

frequency band is composed by two signals at 1028 and 1010 cm^{-1}, mainly due to linear units of different length. Figure S1 b shows the ^{13}C NMR spectrum recorded in $CDCl_3$ on as-synthetized LPMASQ, probing the full hydrolysis of the alkoxide groups and the integrity of propylmethacrylate side-chains. The reproducibility of the sol-gel reaction is also assessed by the ^{29}Si NMR spectrum (Figure S1c), which displays the resonances of T^3 and T^2 different components, whose ratio is in accordance with previous results.

A

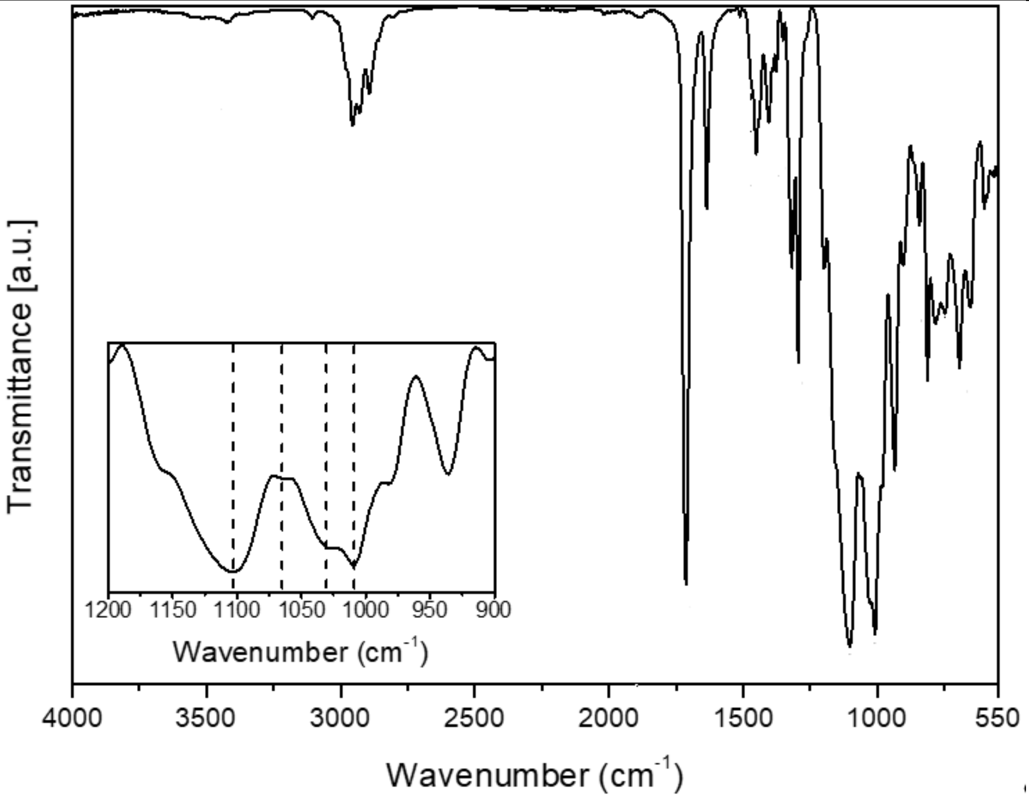

B

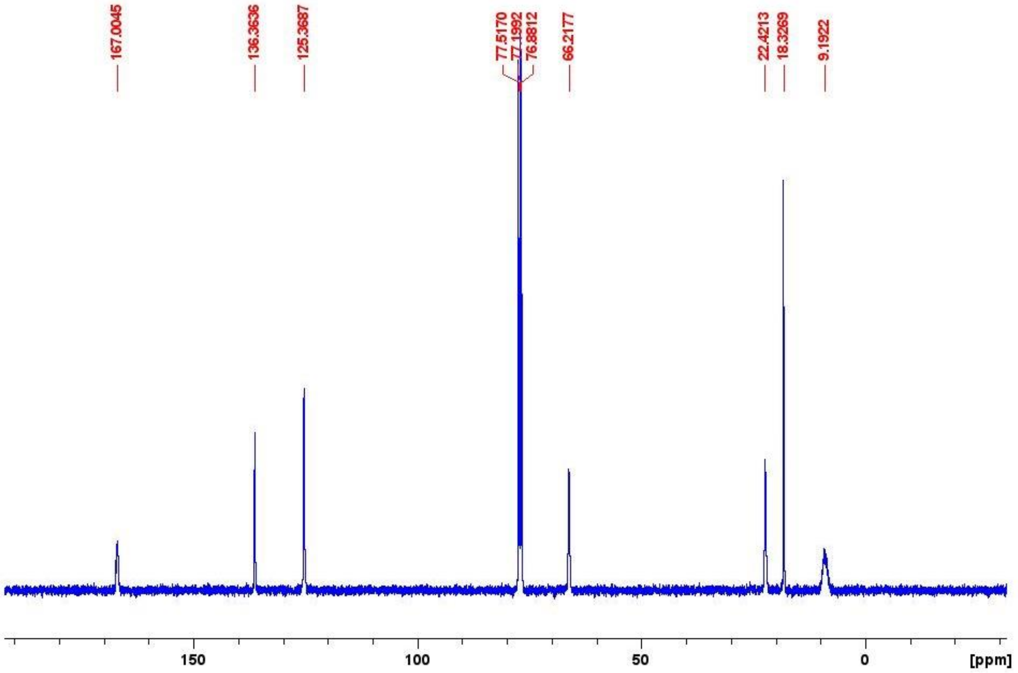

C

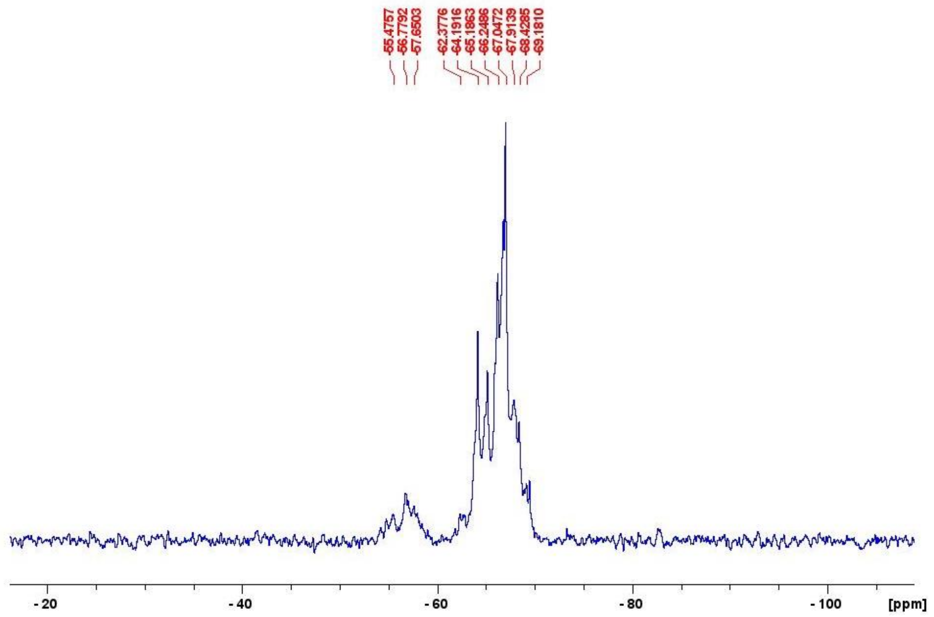

Figure S1. A) ATR-FTIR spectrum, B) ^{13}C NMR and C) ^{29}Si NMR spectra in $CDCl_3$ of as-synthetized LPMASQ.

Alumina functionalization

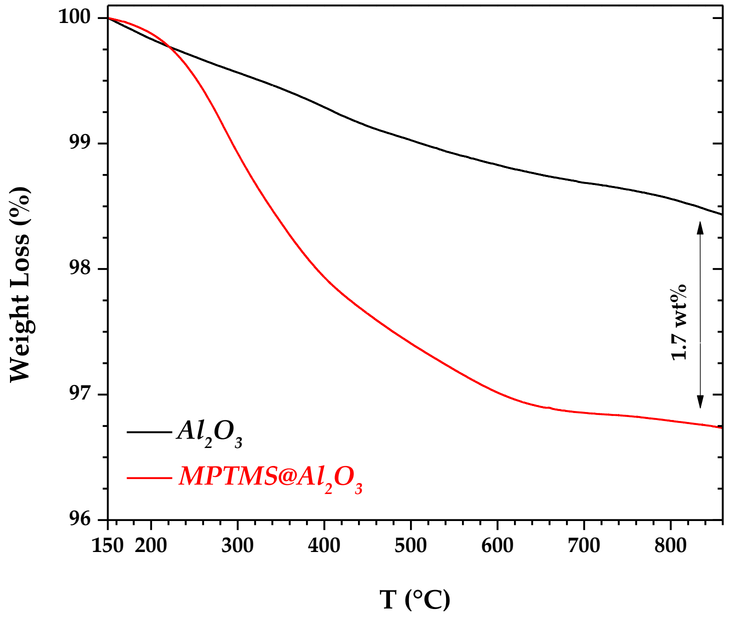

Figure S2. TG analyses of bare (black) and functionalized (red) alumina nanoparticles.

The yield of particles functionalization was evaluated by comparing the TG analyses of bare and functionalized alumina NPs. The amount of methacryloxypropyl trimethoxysilane (MPTMS) grafted

onto the alumina surface was calculated from the net weight loss between 150 and 800 °C, by subtracting the contribution of bare alumina. The grafting density σ was 1.46 molecules · nm^{-2}, calculated according to the following Equation:

$$\sigma = \frac{m_{sil} \cdot N_A}{MW_{sil}} \cdot \frac{1}{m_{NPs} \cdot SSA} = \frac{X_{wt}}{1-X_{wt}} \cdot \frac{N_A}{MW_{sil} \cdot SSA} \quad \text{(S1)}$$

where: m_{sil} = organosilane weight loss; MW_{sil} = organosilane molecular weight; m_{NPs} = mass of employed particles; *SSA* = particles specific surface area [1].

1. Zamperlin, N.; Bottacini, A.; Callone, E.;Pegoretti, A.; Fontana, M. Barium titanate functionalization with organosilanes: Effect on particle compatibility and permittivity in nanocomposites. *Molecules* **2022**, 27, 6499. https://doi.org/10.3390/molecules27196499

LPMASQ/alumina nanocomposites

The volume fraction of the nanocomposites (vol%) has been calculated according to Equation S2:

$$vol\% = 1 - vf - \left(\frac{\rho_{NC}}{\rho_L}\,(1 - w_A\right) \quad \text{(S2)}$$

Where ρ_{NC} is the experimental density of the nanocomposite (Table S3), ρ_L is the density of LPMASQ, w_A is the weight fraction of bare or modified alumina, and vf is the void fraction, calculated according to Equation S3:

$$vf = \frac{\rho_{theo} - \rho_{NC}}{\rho_{theo}} \quad \text{(S3)}$$

Where ρ_{theo} is the theoretical density of the nanocomposite, calculated according to Equation S4:

$$\rho_{theo} = \frac{1}{\frac{w_A}{\rho_A} + \frac{1-w_A}{\rho_L}} \quad \text{(S4)}$$

Where ρ_A= 3.95 g cm^{-3}, is the density of bare and modified alumina.

Table S1. Degree of polymerization (DP) calculated from the integration of d and d' resonances in ^{13}C CPMAS NMR spectra of LPMASQ/alumina nanocomposites.

**Sample**	**DP % (±1)**	**sample**	**DP % (±1)**
L	67	10AF_L	67
10A_L	68	20AF_L	66
20A_L	61	40AF_L	63
40_L	60	80AF_L	60
		120AF_L	60

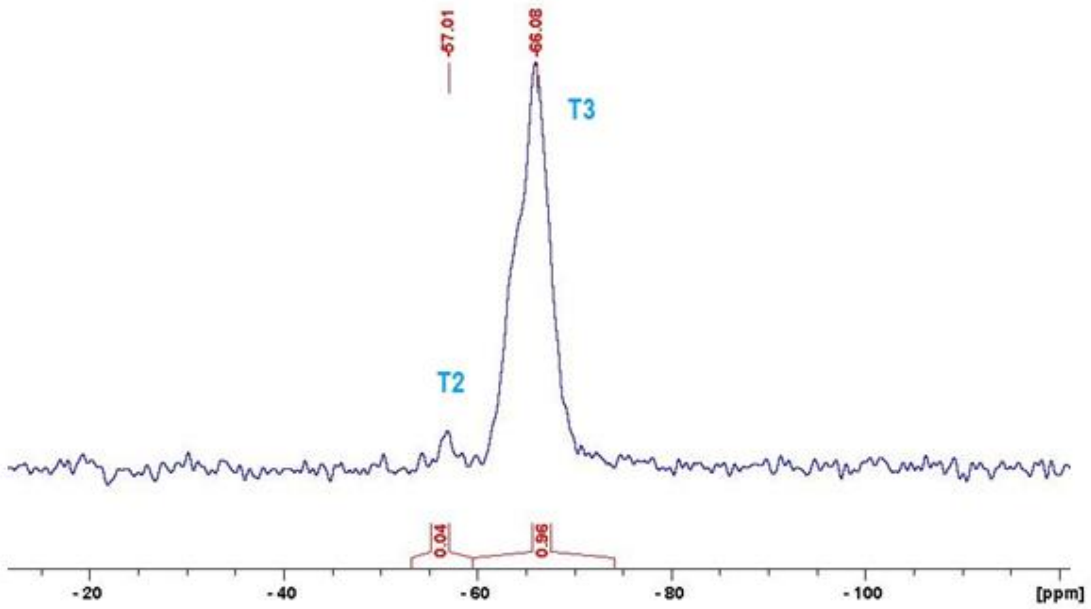

Figure S3. ^{29}Si CPMAS NMR spectrum of photo-cured L sample

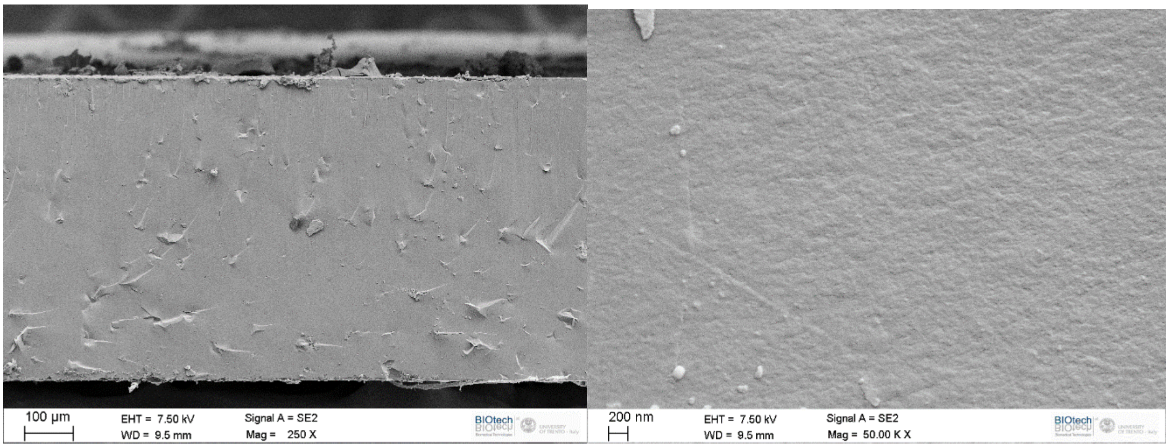

Figure S4: SEM cross section image of L sample at high and low magnification

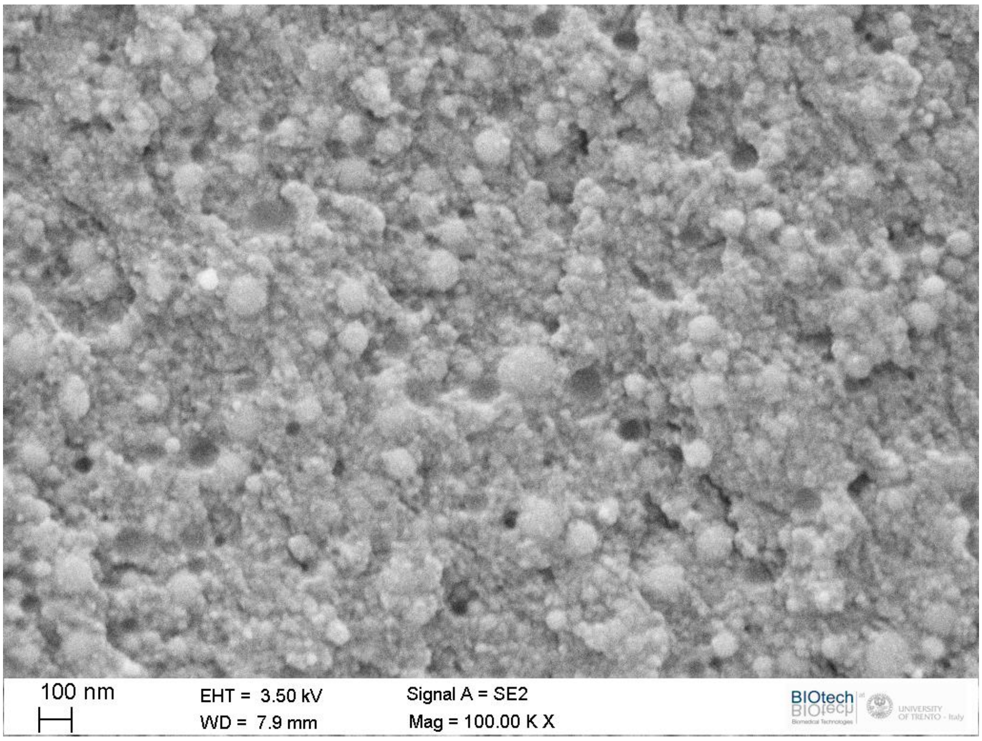

Figure S5: SEM cross-section image of sample 80AF_L

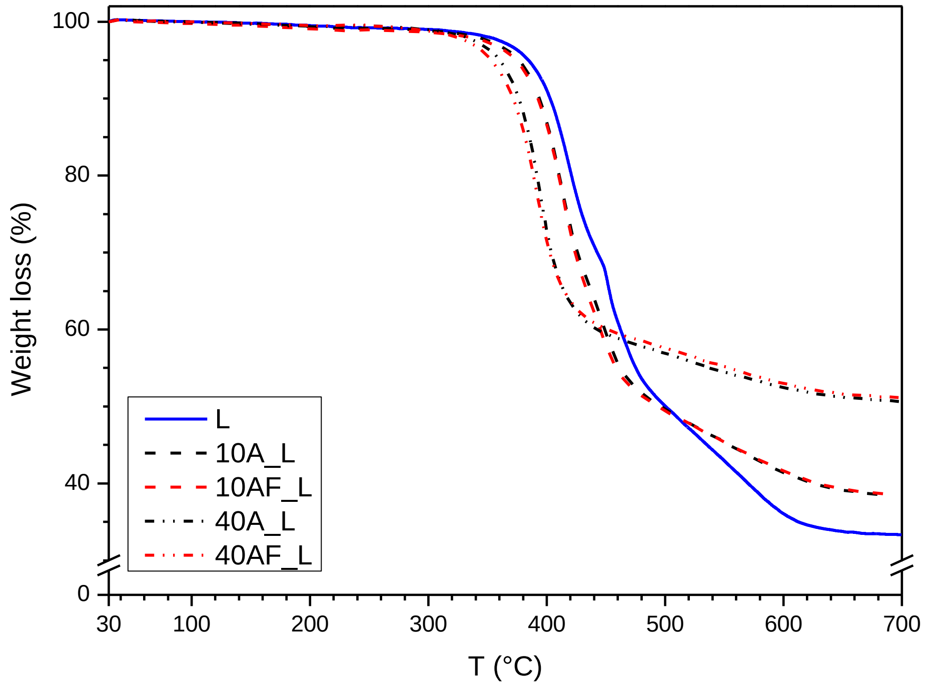

Figure S6. TGA curves of photo-cured LPMASQ (blue) and LPMASQ/alumina nanocomposites with both bare (black) and functionalized NPs (red).

Table S2. Results of TG analyses of LPMASQ/alumina and PB/LPMASQ/alumina nanocomposites: decomposition temperatures T5 and T20, correspond to the temperature at which the sample weight losses are 5% and 20%, respectively; the residual mass is calculated at 700°C.

**Sample**	**T5 (°C)**	**T20 (°C)**	**Residual mass (wt.%)**	**sample**	**T5 (°C)**	**T20 (°C)**	**Residual mass (wt. %)**
L	385	421	33.3	PB	430	451	0
10A_L	376	410	38.4	PB/80AF_10L	427	449	8
20A_L	378	410	43.9	PB/205AF_10L	437	459	17
40_L	360	392	50.7	PB/512AF_25L	422	454	31
10AF_L	374	410	38.6	PB/512AF_40L	401	451	29
20AF_L	373	407	43.8	PB/800AF_40L	389	452	37
40AF_L	353	389	51.1				

PB/LPMASQ/alumina nanocomposites

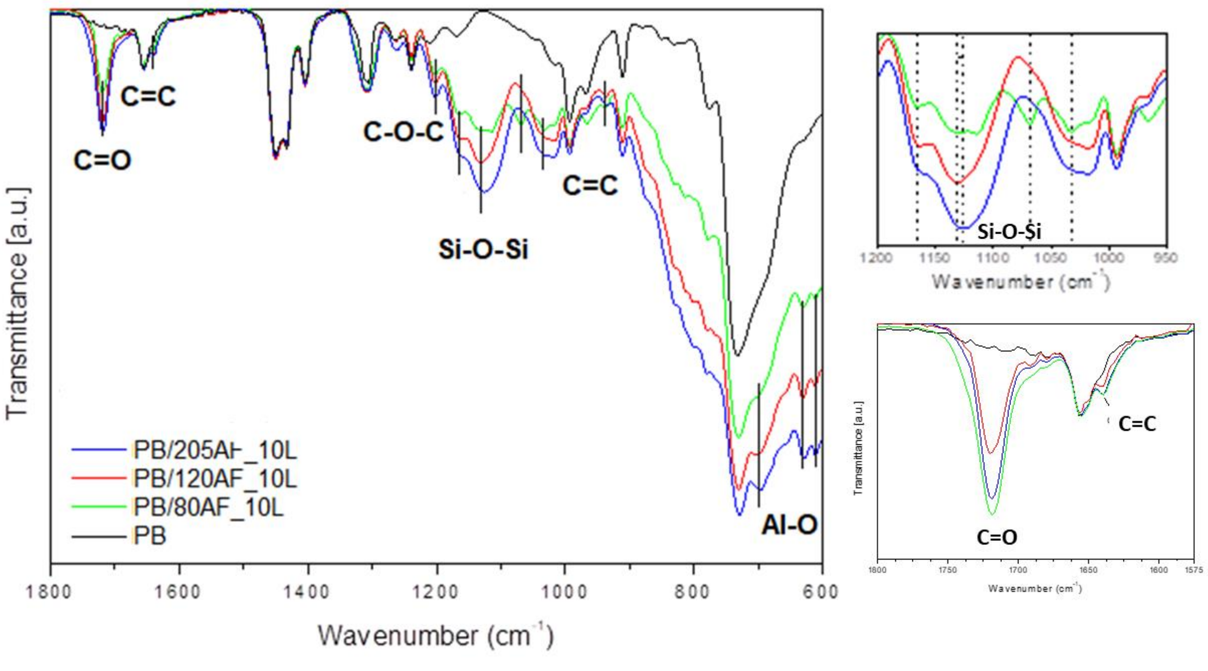

Figure S7. ATR-FTIR spectra of PB/LPMASQ/AF nanocomposites superimposed to the spectrum of PB; the signals of LPMASQ and modified nanoparticles are highlighted. On the right: magnifications of both the siloxane band in the 1200 – 950 cm^{-1} range and the C=O and C=C stretching vibrations.

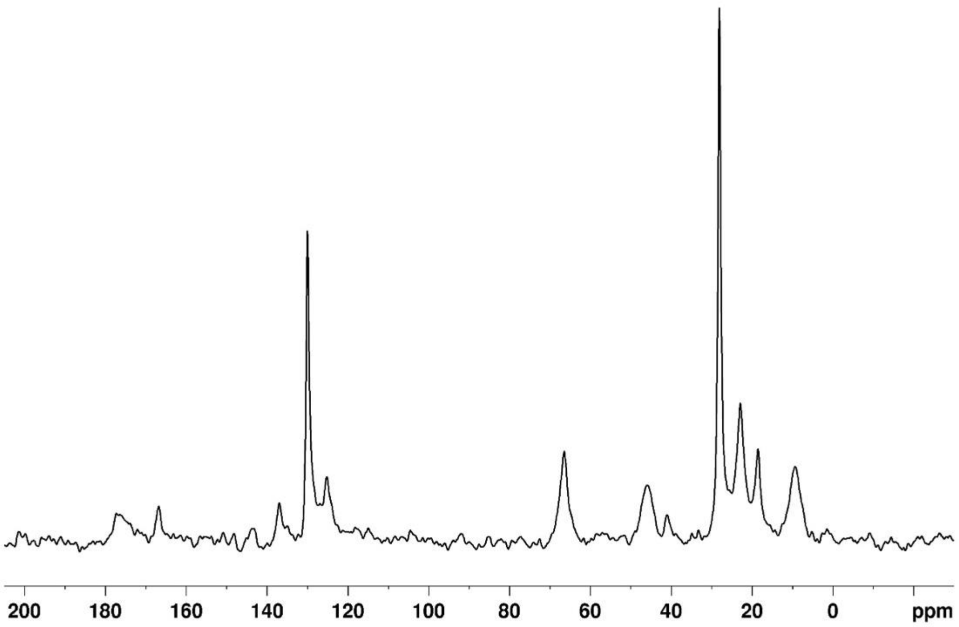

Figure S8. ^{13}C CPMAS NMR spectrum of PB/120AF_10L nanocomposite, as a representative sample.

The carbon spectrum of PB/120AF_10L nanocomposite (Figure S9) shows the typical resonances of both PB and LPMASQ. Unfortunately, the high molecular mobility of cis PB does not allow a proper CPMAS spectrum to be recorded. Anyway, together with the resonances of partially polymerized LPMASQ (Figure 2) and MPTMS-functionalized particles, the two main narrow signals (129.5 and 27.6 ppm), respectively, due to =CH and −CH_2− functional groups in cis-1,4-configuration of commercial polymer PB are visible. The sharpness of these resonances is due to the high mobility of the functional groups, leading to the averaging of nearly all dipolar and chemical shift interactions. 1,2-unit (v) and trans-1,4-units (t) are detectable from weak peaks and account for 5% of the total units: the methyne carbon resonance in 1,2-units (v) occurs at 41 ppm, while the olefinic carbons of 1,2-units give rise to signals at −114 (=CH_2) and −142 (−CH=) ppm.

Thermal conductivity of nanocomposites

Table S3. Density, specific heat, thermal diffusivity, and thermal conductivity values of the prepared samples.

Samples	Density[§] (g cm^{-3})	Specific heat* (J g^{-1} K^{-1})	Thermal diffusivity (mm^2 s^{-1})	Thermal conductivity (W m^{-1} K^{-1})
**L**	1.23 ± 0.01	3.81 ± 0.26	0.146 ± 0.001	0.68 ± 0.05
**10A_L**	1.30 ± 0.02	3.89	0.143 ± 0.001	0.72 ± 0.05
**20A_L**	1.36 ± 0.02	3.95	0.159 ± 0.001	0.85 ± 0.05
**40A_L**	1.47 ± 0.02	4.05	0.186 ± 0.001	1.11 ± 0.06
**10AF_L**	1.32 ± 0.01	3.71	0.142 ± 0.001	0.70 ± 0.05
**20AF_L**	1.37 ± 0.02	3.63	0.159 ± 0.001	0.79 ± 0.05
**40AF_L**	1.46 ± 0.03	3.50	0.191 ± 0.001	0.98 ± 0.06
**80AF_L**	1.70 ± 0.01	3.33	0.216 ± 0.001	1.22 ± 0.06
**120AF_L**	1.82 ± 0.02	3.22	0.236 ± 0.001	1.38 ± 0.06
**PB**	0.81 ± 0.01	2.19 ± 0.11	0.105 ± 0.001	0.18 ± 0.01
**PB_10L**	0.99 ± 0.01	2.36	0.080 ± 0.001	0.19 ± 0.01
**PB/80AF_10L**	0.86 ± 0.01	2.38	0.109 ± 0.001	0.22 ± 0.01
**PB/120AF_10L**	0.86 ± 0.01	2.39	0.121 ± 0.001	0.25 ± 0.01
**PB/205AF_10L**	0.86 ± 0.01	2.42	0.138 ± 0.001	0.28 ± 0.01
**PB_25L**	1.00 ± 0.01	2.60	0.080 ± 0.001	0.21 ± 0.01
**PB/512AF_25L**	1.29 ± 0.01	2.64	0.125 ± 0.001	0.42 ± 0.01
**PB_40L**	1.03 ± 0.01	2.84	0.077 ± 0.001	0.22 ± 0.01
**PB/512AF_40L**	1.11 ± 0.01	2.81	0.127 ± 0.001	0.39 ± 0.01
**PB/800AF_40L**	1.21 ± 0.01	2.80	0.139 ± 0.001	0.47 ± 0.01

*The specific heat values of L and PB have been measured as described in the experimental Section. The specific heat values of the nanocomposites have been calculated according to Eq. 2 (see main text).

§ Some deviations from the expected trends of the density values are possibly due to local inhomogeneity, nanoporosity and voids around the filler particles in the nanocomposites. In perspective, further refining the processing could result in higher quality of the nanocomposites.

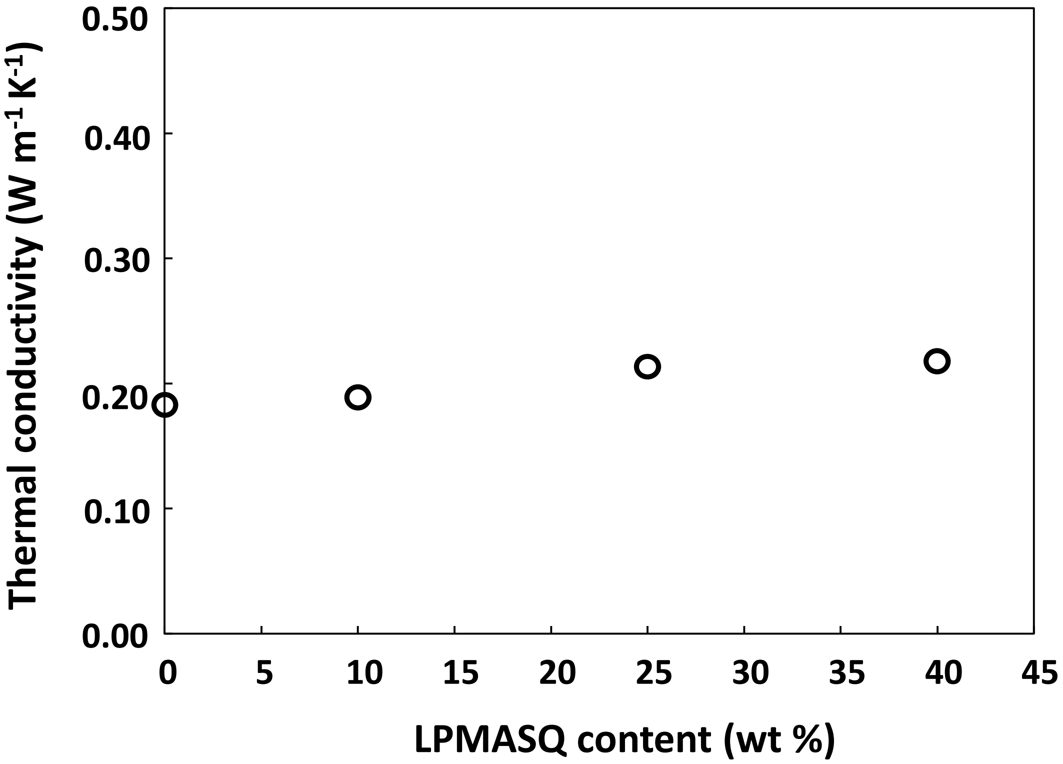

Figure S9. Thermal conductivity values of nanocomposites containing PB and different amounts of LPMASQ
